# Use of Texture Analysis on Noncontrast MRI in Classification of Early Stage of Liver Fibrosis

**DOI:** 10.1155/2021/6677821

**Published:** 2021-03-18

**Authors:** Ru Zhao, Xi-Jun Gong, Ya-Qiong Ge, Hong Zhao, Long-Sheng Wang, Hong-Zhen Yu, Bin Liu

**Affiliations:** ^1^Department of Radiology, The First Affiliated Hospital of Anhui Medical University, 218 Jixi Road, Hefei 230022, Anhui, China; ^2^Department of Radiology, The Second Affiliated Hospital of Anhui Medical University, 678 Furong Road, Hefei 230601, Anhui, China; ^3^GE Healthcare China, Pudong New Town, No. 1, Huatuo Road, Shanghai 210000, China; ^4^Department of Pathology, The Second Affiliated Hospital of Anhui Medical University, 678 Furong Road, Hefei 230601, Anhui, China

## Abstract

*Purpos*e. To compare the diagnostic value of texture analysis- (TA-) derived parameters from out-of-phase T1W, in-phase T1W, and T2W images in the classification of the early stage of liver fibrosis. *Methods*. Patients clinically diagnosed with hepatitis B infection, who underwent liver biopsy and noncontrast MRI scans, were enrolled. TA parameters were extracted from out-of-phase T1-weighted (T1W), in-phase T1W, and T2-weighted (T2W) images and calculated using Artificial Intelligent Kit (AK). Features were extracted including first-order, shape, gray-level cooccurrence matrix, gray-level run-length matrix, neighboring gray one tone difference matrix, and gray-level differential matrix. After statistical analyses, final diagnostic models were constructed. Receiver operating curves (ROCs) and areas under the ROC (AUCs) were used to assess the diagnostic value of each final model and 100-time repeated cross-validation was applied to assess the stability of the logistic regression models. *Results*. A total of 57 patients were enrolled in this study, with 27 in the fibrosis stage < 2 and 30 in stages ≥ 2. Overall, 851 features were extracted per ROI. Eight features with high correlation were selected by the maximum relevance method in each sequence, and all had a good diagnostic performance. ROC analysis of the final models showed that all sequences had a preferable performance with AUCs of 0.87, 0.90, and 0.96 in T2W and in-phase and out-of-phase T1W, respectively. Cross-validation results reported the following values of mean accuracy, specificity, and sensitivity: 0.98 each for out-of-phase T1W; 0.90, 0.89, and 0.90 for in-phase T1W; and 0.86, 0.88, 0.84 for T2W in the training set, and 0.76, 0.81, and 0.72 for out-of-phase T1W; 0.74, 0.72, and 0.75 for in-phase T1W; and 0.63, 0.64, and 0.63 for T2W for the test group, respectively. *Conclusion*. Noncontrast MRI scans with texture analysis are viable for classifying the early stages of liver fibrosis, exhibiting excellent diagnostic performance.

## 1. Introduction

Liver fibrosis is the pathological repair response to chronic liver disease and the key step in the development of cirrhosis, characterized by excessive accumulation and abnormal distribution of extracellular matrix [1]. Hepatitis B is becoming a major global health crisis since WHO estimated in 2015 that almost 260 million people were living with chronic hepatitis B virus infection, resulting in more than 800,000 deaths annually [2]. Patients with hepatitis B virus infection are at risk of progression to cirrhosis, which is often accompanied by many complications such as hepatocellular carcinoma and is strongly associated with mortality and low quality of life. With the development of medicine, some reports found that cirrhosis could be prevented or reversed if therapy was administered in the early stages of liver fibrosis [3–6] making the diagnosis of the early stage of liver fibrosis critical. According to guidelines on the management of hepatitis B virus infection [7, 8], one of the treatment options that stage 2 or higher of liver fibrosis will be necessary to accept the antiviral therapy is the classification standard of this study. Liver biopsy, the current reference standard for the assessment of liver fibrosis, is an invasive method with some known complications including bleeding, pain, sample errors, and interobserver variability [9]. Alternative noninvasive methods that can substitute liver biopsy are still under investigation. MRI has high sensitivity in the detection of nodular liver parenchymal changes in cirrhosis but has a poor visual assessment of liver fibrosis, especially in the early stage. Thus, texture feature extraction and machine learning based on MRIs have been suggested. Texture analysis (TA) is a new useful, postprocessing method that can provide more data than the perceivable texture feature parameters. These texture features can be extracted through machine learning software and can reflect the extent of heterogeneity, granularity, randomness, and so forth, which may be associated with histopathological changes and contribute to the differential diagnosis and assessment of the development stage of fibrosis. Several promising studies have reported that texture analysis based on MR images can be used for the classification of liver fibrosis [10, 11], especially in advanced fibrosis and significant cirrhosis. Few studies have reported methods of classification for the early stages of liver fibrosis, for early detection and subsequent early treatment that can help prevent its progression and ultimately reduce the occurrence of complications related to chronic liver disease.

The aim of this study was to construct diagnostic models by analyzing TA-derived imaging parameters based on noncontrast MR images, including out-of-phase T1-weighted (T1W), in-phase T1-weighted (T1W), and T2-weighted (T2W) images of early stages of liver fibrosis and compare their diagnostic performance to explore whether noncontrast MR images can be effective in classifying the early stages of liver fibrosis.

## 2. Materials and Methods

This retrospective study was approved by the institutional review board and local ethics committee. Written informed consent was obtained from all study participants. All liver biopsies were clinically indicated.

### 2.1. Study Participants

Participants who were clinically diagnosed with hepatitis B infection and underwent both MRI scan and liver biopsy were screened for this study between July 2016 and September 2019. The standard parameters of diagnosis of hepatitis B infection are the presence of hepatitis B surface antigen in the serum and a repeat positive after 6 months. Inclusion criteria were as follows: (1) adults patients 18 years of age or older; (2) patients who were diagnosed with hepatitis B clinically and had no other coexisting chronic liver diseases; (3) patients who underwent MRI scans before undergoing liver biopsies; (4) patients who are willing to participate in this study and signed a written informed consent form; and (5) absence of any signs of cirrhosis on MRI scans. Exclusion criteria were as follows: (1) patients with claustrophobia; (2) MR images with large respiration or motion artifacts; (3) decompensated cirrhosis; and (4) definite cirrhosis during the imaging and pathological diagnosis. A total of 58 participants were screened: 57 were included while one was excluded due to the failure of TA extraction.

### 2.2. MRI Data Acquisition of Liver

All MRI scans were performed on the same 1.5 T clinical system (Avanto, Siemens Healthcare, Erlangen, Germany) using a 4-channel body phased-array coil. All participants underwent the same abdominal MRI protocol, which consisted of the following sequences: in-phase and out-of-phase T1-weighted axial images and T2-weighted fat-saturated axial images. The imaging parameters of the T1W sequence were TR (repetition time) 200 ms, TE (time to echo) 2.2 ms/4.4 ms (in-phase/out-of-phase), averages 1, concatenations 1, FoV (field of view) read 380 mm, and FoV phase 78.1%, and slice thickness 6.0 mm and T2W sequence was TR 4000 ms, TE 79 ms, averages 1, concatenations 1, FoV read 400 mm, FoV phase 70.3%, and slice thickness 6.0 mm.

### 2.3. Histological Analysis

Liver biopsy was performed under ultrasound guidance. Histopathologic features were evaluated by a pathologist with 10 years of experience who was blinded to patients' MRI diagnoses. The biopsy sampling area was selected in segment V or VIII, according to Couinaud's liver segmentation [12, 13]. The fibrosis stages were assessed according to the METAVIR scoring system [14] and standardized to the common scale. Standardization was as follows: F0, no fibrosis; F1, portal fibrosis without septa; F2, portal fibrosis with rare septa; F3, numerous septa without cirrhosis; and F4, cirrhosis.

In our study, stages F0–F1 of fibrosis were categorized as early-stage fibrosis and stages F2–F4 as significant fibrosis. According to guidelines on the management of hepatitis B virus infection, [7, 8] patients with fibrosis stages ≥ 2 need antiviral therapy. The enrolled participants were classified into two groups: patients with early stages of fibrosis who did not require antiviral therapy and patients in the significant fibrosis group who required antiviral therapy.

### 2.4. Acquisition of Texture Features

**Figure 1:**
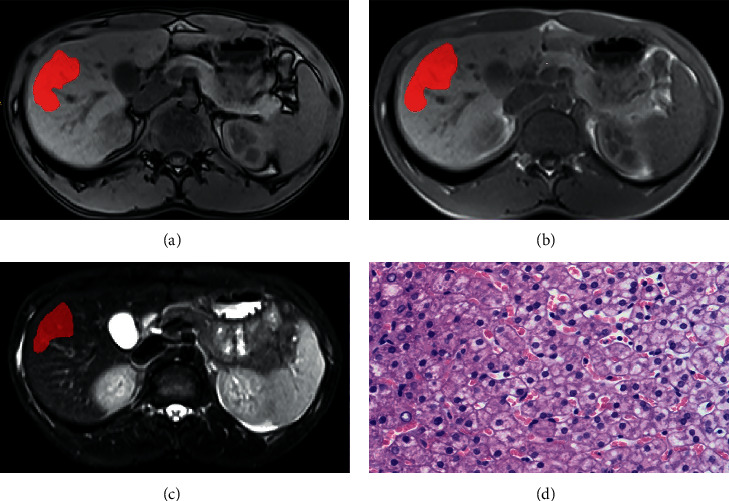
A 19-year-old man with hepatitis B infection. (a–c) ROI delineated using ITK-SNAP on out-of-phase T1W, in-phase T1W, and T2W images. (d) Histopathological features obtained from liver biopsy revealed stages 0-1 of fibrosis. The patient was classified in the low-stage fibrosis by the final models.

**Figure 2:**
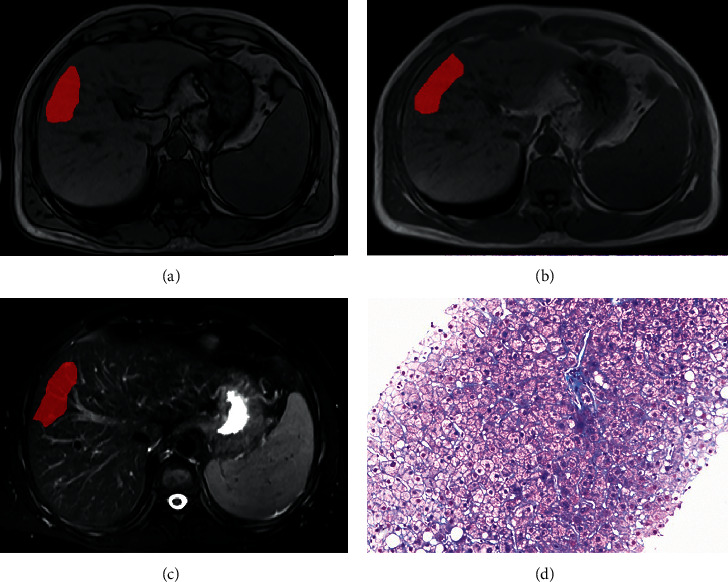
A 40-year-old man with hepatitis B infection. (a–c) ROI delineated using ITK-SNAP on out-of-phase T1W, in-phase T1W, and T2W images. (d) Histopathological features obtained from liver biopsy revealed stage 2 fibrosis. The patient was classified in significant fibrosis by the final models.

**Figure 3:**
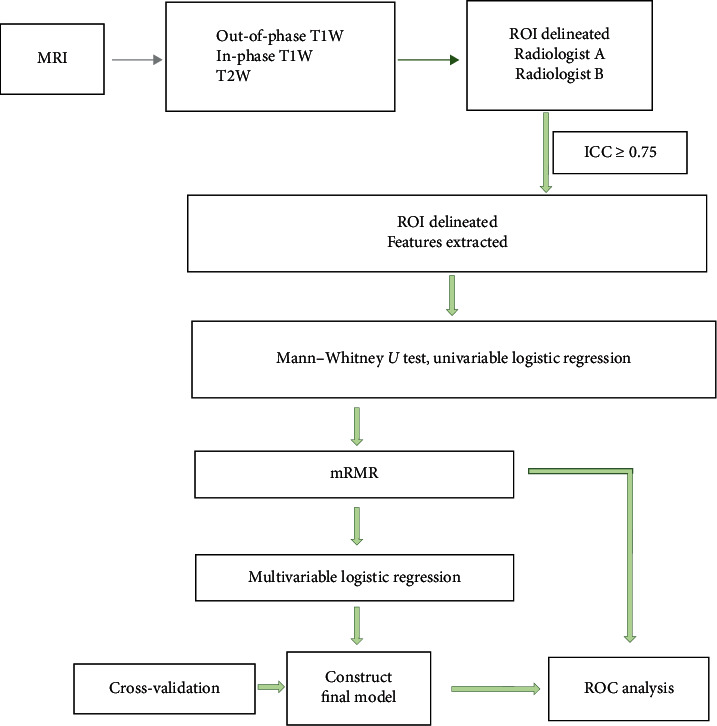
Flowchart of feature selection and texture analysis.

Texture features were extracted from in-phase and out-of-phase T1WI as well as T2WI images of all participants by two radiologists (with 8 and 5 years of experience in abdominal imaging diagnosis, respectively). All participants' images were exported in DICOMS format and then imported into an open-source software program (ITK-SNAP, V3.30) [15] for manual receiver of interest (ROI) delineation. For each sequence, one slice from the biopsy sampling area with no liver lesions and a low amount of motion artifacts was selected. A free-hand ROI, as large as possible and avoiding major blood vessels or liver lesions, was placed on T1WI and T2WI. All DICOMS and ROI images were imported into A.K (Artificial Intelligence Kit Version; V3.2.0 R, GE Healthcare, Shanghai). Data processing steps were as follows: the linear interpolation method was resampled with dimensions 1 ∗ 1 ∗ 1 mm. Gaussian filter function was denoised and gray-level discretized and normalized, reconstructed with Z-score standardization (mean of 0 and deviation of 1), and fused. Extracted features based on ROI included the first-order, shape, gray-level cooccurrence matrix, gray-level run-length matrix, neighboring gray one tone difference matrix, and gray-level differential matrix and with the features' transform type included. Overall, 851 texture features were extracted per ROI (Figures 1–3).

### 2.5. The Intraobserver and Interobserver Agreements

The intraclass correlation coefficient (ICC) was applied to analyze the intraobserver and interobserver agreements of the feature extraction. Out-of-phase T1W images of all participants were used for exploring interobserver ICC, while T2W images were used for intraobserver ICC. Radiologist A delineated the ROIs twice on two different weeks to evaluate the intraobserver agreement and Radiologist B independently delineated them once to evaluate the interobserver agreement with the ROIs delineated by Radiologist A. Features with mean values of the intra- and interclass ICC higher than 0.75 were retained [16].

### 2.6. Statistical Analysis

During the texture analysis process, the data were analyzed using the following procedure: (1) Mann–Whitney *U* test, to explore whether the features were significant (*p* < 0.05); (2) univariate logistic regression, to explore whether the features were discriminative between the two groups (*p* < 0.05). (3) the minimum redundancy and maximum relevance (mRMR) method, to select features without redundancy and with high correlation; (4) stepwise multivariable logistic regression, to construct the predictive model, features with *p* < 0.05 being independently discriminative; and (5) 100-time repeated cross-validation to prove that the logistic model was valuable in discriminating one group from another, in consideration of the small number of datasets, and that the result was not due to overfitting (Figure 3). Receiver operating curves (ROC) and areas under the ROC (AUC) were used to assess the diagnostic value of each selected feature and final model.

## 3. Results

57 participants (20 women and 37 men) were enrolled in this study: 27 having early-stage fibrosis and 30 with significant fibrosis (details are shown in Table 1).

The intraobserver ICC of the same reader ranged from −0.31 to 0.99, and the interobserver ICC of two independent readers ranged from −0.21 to 0.95. A total of 126 features were retained and they showed high intra- and interobserver ICC with ICCs ≥ 0.75. Thus, features extracted by Radiology A were used for further analysis.

### 3.1. Texture Analysis

**Figure 4:**
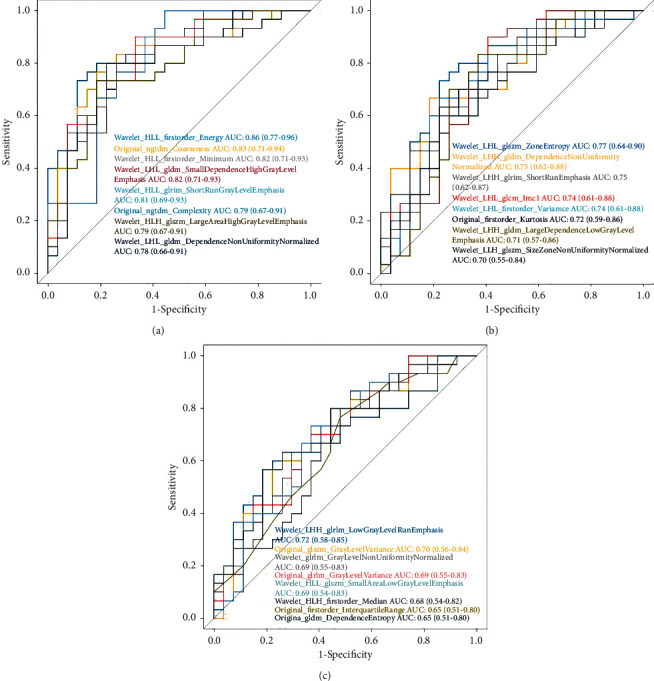
AUC of the selected features with high correlation of each sequence (out-of-phase T1W, in-phase T1W, and T2W).

After univariate logistic regression to select discriminative features, 8 texture features without redundancy and with high correlations with labels were selected from 851 original parameters from the in-phase T1W, out-of-phase T1W, and T2W images. Each feature showed a good result (Figure 4). The performance of these features is shown in Table 2.

### 3.2. Model Construction

**Figure 5:**
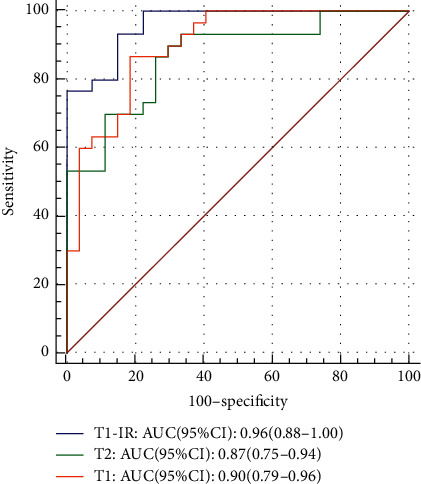
AUC of the final model of each sequence (T1-IR: out-of-phase T1W, T1: in-phase T1W, and T2 : T2W).

The most predictive features were selected and the final model was constructed in accordance with the mRMR method through backward stepwise selection with the likelihood radio test in the in-phase T1W, out-of-phase T1W, and T2W images. The ROC analysis of the final model showed that all the sequences had a preferable performance with AUC greater than 0.87, while the DeLong test showed that no significant difference was found among each sequence (Figure 5). Therefore, we concluded that ROC analysis of the final models can be used in the differentiation of the early-stage and significant fibrosis.

The 100-time repeated cross-validation was applied to assess the stability of the logistic regression models. Patients' data were randomly classified into training and validation set at a ratio of 7 : 3. Results of the 100-time cross-validation are shown in Table 3. Cross-validation results gave a mean accuracy, specificity, and sensitivity of 0.98 each for out-of-phase T1W; 0 90, 0.89, and 0.90 for in-phase T1W; and 0.86, 0.88, and 0.84 for T2W in the training set and values of 0.76, 0.81, and 0.72 for out-of-phase T1W; 0.74, 0.72, and 0.75 for in-phase T1W; and 0.63, 0.64, and 0.63 for T2W in the test group (Table 3). It revealed that the out-of-phase T1W images had the best accuracy and stability among these three sequences.

## 4. Discussion

The diagnostic tool is defined as perfect if the AUC is 100%, excellent if it is greater than 90%, and good if it is greater than 80% [17]. According to this, the ROC analysis of diagnosis models based on TA parameters derived from out-of-phase T1W, in-phase T1W, and T2WI images in this study all exhibited good diagnostic accuracy. Cross-validation also reported good diagnostic accuracy in the training set, fair-to-good for T1W images and poor for T2W images in the test group. The leading cause maybe is data instability related to the limited numbers of participants. Therefore, a larger number of patients would have been required in the next study. Even though there was no statistical difference among TA parameters derived from the three sequences, AUC results showed that TA from out-of-phase T1WI exhibited the best performance in the differentiation of early-stage fibrosis (F0–F1) from significant fibrosis (F2–F4). The possible reason may be the attenuation of the fat signal in out-of-phase T1W images and it is interesting to note that steatosis, which shows diffusely decreased liver attenuation, is also one of the features of chronic liver disease. Some studies have shown that TA extracted from T1W images has an excellent performance in the classification of liver fibrosis [10, 11]. However, our study may be the first to explore TA from out-of-phase T1W images to classify early-stage fibrosis and to obtain better AUC results compared to TA from in-phase T1W and T2W images.

The classification of liver fibrosis using TA features derived from MR images is under investigation. In 2002, Daniel [18] reported that TA based on T2W images can be successfully used for separating cirrhotic patients and healthy volunteers. Subsequently, an increasing number of reports on TA-derived parameters from MR images for classifying liver fibrosis have been published. Wang [19] et al. demonstrated that diffusion-weighed imaging (DWI) had a diagnostic accuracy of 0.86, 0.83, and 0.86 for stages 1, 2, and 3 of liver fibrosis, respectively, which was lower than the accuracy of magnetic resonance elastography (MRE), through a meta-analysis of 14 published reports. Jiang et al. [20] also revealed that DWI exhibited good diagnostic accuracy for classifying fibrosis stages and that a higher *b* value can optimize diagnostic performance, through a meta-analysis of 12 published reports. House et al. [21] revealed that texture features derived from T2W images demonstrated diagnostic sensitivity for discriminating patients with or without fibrosis with an AUC of 0.78 but was less sensitive in staging low and intermediate levels of fibrosis. Yokoo et al. [22] reported that TA based on combined-contrast-enhanced (CCE) MR images could be quantified to predict fibrosis severity by analyzing 165 texture features extracted from MR images of 46 HCV-infected patients who underwent CCE liver MRI with the administration of superparamagnetic iron oxides and gadolinium DTPA. Cannella et al. [11] analyzed five histogram-based parameters extracted on noncontrast 3D-GRE T1W images in 54 patients with nonalcoholic fatty liver disease and concluded that standard deviation and entropy were positively correlated with the degree of liver fibrosis with AUC 0.755 and 0.769 for significant fibrosis (F2–F4) and 0.746 and 0.754 for advanced fibrosis (F3–F4), respectively. Recently, Schawkat et al. [10] reported that the TA parameters of T1W images demonstrated accuracy similar to that of MRE. They analyzed 308 texture features extracted from T1W and T2W images of 62 patients and compared them to those of the MRE results and then assessed the diagnostic accuracy of the classification of liver fibrosis in low-stage (F0–F2) and high-stage (F3–F4) fibrosis. They proved that TA derived from T1W images had better diagnostic accuracy than T2W images which was consistent with our findings.

Noninvasive methods of diagnosing liver fibrosis include serologic markers, MRE, and transient elastography (TE). A single serum induced marker has limited function in the assessment of fibrosis, but a combination of testing has an improved diagnostic value. A previous study had reported that combinational serologic models could substitute liver biopsy to a certain extent and this reduces the need for liver biopsy by 30–40%, but they were effective only in distinguishing between the absence of fibrosis and advanced fibrosis [23]. MRE and TE have good diagnostic performance and clinical applicability among all the noninvasive diagnostic methods of liver fibrosis [24, 25]. Compared to MRE, TE is a much quicker and less expensive noninvasive method for assessing liver stiffness, although it needs a high level of expertise and better diagnostic performance for the classification of advanced fibrosis and cirrhosis, and it also has a high failure rate in obese patients [26]. MRE has a much higher diagnostic accuracy and stability compare to TE. But MRE is more expensive and required a much higher quality of hardware to accomplish this. Compared to the other noninvasive techniques, TA is a quick and easily obtainable method that can be applied retrospectively on routinely acquired images without the need for dedicated hardware. However, TA can be affected by many confounding factors, including ROI size [27], image acquisition and reconstruction parameters, different software packages, and lack of standardized methodology [28]. In this study, for the sake of mitigating some of the variability related to the imaging technique, we applied TA on prospectively acquired MR images on the same MRI scanner with the same scanning parameters.

Our study has some limitations. First, the study population was small due to the inclusion criteria of HBV infection and no morphological changes on CT and MR images. These results need to be confirmed with larger studies. Second, the subjectivity of ROI placement through manual delineation performed on different slices between T1W and T2W and different patients in this study may increase biases. Third, there is a lack of exploring DWI and contrast MR images in this study. The diagnostic models based on T1W and T2W images are too single and may limit clinical practicality. We also had all participants get DWI scans. But we excluded the images in the end because of the large respiration artificial in some patients. Contrast MR scans were excluded at the beginning for the need of injected contrast agent. A further study may be needed to have deep learning in all MRI scans. Fourthly, the lack of stage 0 fibrosis patients included in this study may also lead to range errors. Fifth, even though TA from MRI is quick and accessible, MRI are costly and time-consuming which also limits its clinical application.

## 5. Conclusions

In conclusion, noncontrast MRI scans combined with texture analysis can be used for the classification of early-stage fibrosis (stage < 2 vs. stage ≥ 2) and all had good diagnostic accuracy. And TA parameters extracted from out-of-phase T1W images may have better performance compared to TA based on in-phase T1W and T2W images.

## Figures and Tables

**Table 1:** Distribution of stages of fibrosis.

Stage of fibrosis	Numbers	Categories	Ages
0	0	Early-stage fibrosis	34.15 ± 11.4
1	27		
2	19	Significant fibrosis	42.94 ± 10.5
3	10		
4	1		

**Table 2:** Performance of selected features with high correlation.

Image	Var. name	Threshold	Accuracy	Sensitivity	Specificity	Pos. pred. value	Neg. pred. value
Out-of-phase T1WI	wavelet_HLL_firstorder_Energy	−0.04	0.81 (0.68–0.90)	0.73	0.90	0.88	0.75
	original_ngtdm_Coarseness	−0.35	0.79 (0.66-o.88)	0.77	0.81	0.821	0.76
	wavelet_HLL_firstorder_Minimum	0.21	0.75 (0.62–0.86)	0.77	0.74	0.77	0.74
	wavelet_LHL_gldm_SmallDependenceHighGrayLevelEmphasis	−0.16	0.79 (0.66–0.89)	0.90	0.67	0.75	0.86
	wavelet_HLL_glrlm_ShortRunLowGrayLevelEmphasis	0.43	0.79 (0.66–0.89)	1	0.55	0.71	1
	original_ngtdm_Complexity	−0.27	0.79 (0.66–0.89)	0.80	0.78	0.80	0.78
	wavelet_HLH_glszm_LargeAreaHighGrayLevelEmphasis	−0.28	0.77 (0.64–0.87)	0.73	0.81	0.81	0.73
	wavelet_HLH_gldm_DependenceNonUniformityNormalized	−0.13	0.77 (0.64–0.87)	0.8	0.74	0.77	0.77

In-phase T1WI	wavelet_LHL_glszm_ZoneEntropy	0.16	0.75 (0.62–0.85)	0.73	0.78	0.78	0.72
	wavelet_LHH_gldm_DependenceNonUniformityNormalized	0.18	0.73 (0.60–0.84)	0.67	0.85	0.80	0.68
	wavelet_LHH_glrlm_ShortRunEmphasis	−0.20	0.68 (0.54–0.80)	0.60	0.78	0.75	0.63
	wavelet_LLL_glcm_Imc1	−0.41	0.75 (0.62–0.86)	0.90	0.59	0.71	0.84
	wavelet_LHL_firstorder_Variance	−0.11	0.72 (0.58–0.83)	0.67	0.78	0.77	0.68
	original_firstorder_Kurtosis	−0.57	0.70 (0.56–0.82)	0.83	0.56	0.67	0.75
	wavelet_LHH_gldm_LargeDependenceLowGrayLevelEmphasis	−0.07	0.74 (0.60–0.83)	0.83	0.63	0.71	0.77
	wavelet_LLH_glszm_SizeZoneNonUniformityNormalized	0.08	0.70 (0.56–0.81)	0.63	0.78	0.76	0.65

T2WI	wavelet_LHH_glrlm_LowGrayLevelRunEmphasis	0.20	0.68 (0.55–0.80)	0.54	0.80	0.57	0.81
	original_glszm_GrayLevelVariance	0.16	0.67 (0.53–0.78)	0.53	0.78	0.57	0.78
	original_glrlm_GrayLevelNonUniformityNormalized	−0.30	0.67 (0.53–0.78)	0.53	0.78	0.63	0.70
	original_glrlm_GrayLevelVariance	−0.02	0.67 (0.53–0.78)	0.53	0.78	0.67	0.67
	wavelet_LHL_glszm_SmallAreaLowGrayLevelEmphasis	−0.12	0.68 (0.55–0.80)	0.54	0.80	0.73	0.63
	wavelet_HLH_firstorder_Median	0.38	0.68 (0.55–0.80)	0.54	0.80	0.56	0.81
	original_firstorder_InterquartileRange	−0.36	0.65 (0.51–0.77)	0.51	0.77	0.76	0.52
	original_gldm_DependenceEntropy	−0.06	0.68 (0.55–0.80)	0.54	0.80	0.80	0.56

**Table 3:** Mean value of specificity, sensitivity, and accuracy of 100-time cross-validation in each sequence.

	Group	Specificity	Sensitivity	Accuracy
Out-of-phase T1WI	Training	0.98	0.98	0.98
	Test	0.81	0.72	0.76
In-phase T1WI	Training	0.89	0.90	0.90
	Test	0.72	0.75	0.74
T2WI	Training	0.88	0.84	0.86
	Test	0.64	0.63	0.63

## Data Availability

All patients' data used to support the findings of this study are currently under embargo while the research findings are commercialized. Requests for data (6 months) after the publication of this article will be considered by the corresponding author.
